# Detecting Laterality Errors in Combined Radiographic Studies by Enhancing the Traditional Approach With GPT-4o: Algorithm Development and Multisite Internal Validation

**DOI:** 10.2196/76384

**Published:** 2025-10-29

**Authors:** Kung-Hsun Weng, Yi-Chen Chou, Yu-Ting Kuo, Tsyh-Jyi Hsieh, Chung-Feng Liu

**Affiliations:** 1 Department of Medical Imaging Chi Mei Medical Center Tainan Taiwan; 2 Institute of Precision Medicine College of Medicine National Sun Yat-sen University Kaohsiung Taiwan; 3 Department of Radiology School and College of Medicine Kaohsiung Medical University Kaohsiung Taiwan; 4 Department of Medical Research Chi Mei Medical Center Tainan Taiwan

**Keywords:** natural language processing, large language model, radiographic reports, laterality error, radiology report, quality assurance, rule-based methods, artificial intelligence, deep learning, electronic health records

## Abstract

**Background:**

Laterality errors in radiology reports can endanger patient safety. Effective methods for screening for laterality errors in combined radiographic reports, which combine multiple studies into one, remain unexplored.

**Objective:**

First, we define and analyze the unstudied combined radiographic report format and its challenges. Second, we introduce a clinically deployable ensemble method (rule-based+GPT-4o), evaluated on large-scale, real-world, imbalanced data. Third, we demonstrate significant performance gaps between real-world imbalanced and synthetic balanced datasets, highlighting limitations of the benchmarking methodology commonly used in current studies.

**Methods:**

This retrospective study analyzed deidentified English radiology reports containing laterality terms in order. We split the data into TrainVal (combined training and validation dataset), Test-1 (both real-world, imbalanced), and Test-2 (synthetic, balanced). Test-1 comes from a distinct branch. Experiment 1 compared the baseline, workaround, and GPT-4o-augmented rule-based methods. Experiment 2 compared the rule-based method with the highest recall to fine-tuned RoBERTa, ClinicalBERT, and GPT-4o models.

**Results:**

As of July 2024, our dataset included 10,000 real-world and 889 synthetic radiology reports. The laterality error rate in real-world reports was 1.20% (120/10,000), significantly higher in combined (103/7000, 1.47%) than in noncombined reports (17/3000, 0.57%; difference=0.90%; *z*=3.81; *P*<.001). In experiment 1, recall differed significantly among the 3 versions of rule-based methods (*Q*=6.0; *P*=.0498, Friedman test). The rule-based+GPT-4o method had the highest recall (average rank=1), significantly better than the baseline (average rank=3; *P*=.04, Nemenyi test). Most (5/6) of the false positives introduced by the GPT-4o information extraction were due to parser limitations hidden by error cancellation. In experiment 2, on Test-1, rule-based+GPT-4o (precision=0.696; recall=0.889; *F*_1_-score=0.780) outperformed GPT-4o (precision=0.219; recall=0.889; *F*_1_-score=0.352), ClinicalBERT (precision=0.047; recall=0.667; *F*_1_-score=0.088), and RoBERTa (*F*_1_-score=0.000). On Test-2, rule-based+GPT-4o (precision=0.996; recall=0.925; *F*_1_-score=0.959) and GPT-4o (precision=0.979; recall=0.953; *F*_1_-score=0.966) outperformed ClinicalBERT (precision=0.984; recall=0.749; *F*_1_-score=0.851) and RoBERTa (*F*_1_-score=0.013). Both ClinicalBERT and GPT-4o exhibited notable declines in precision on TrainVal and Test-1 compared to Test-2. Both Test-1 data membership (GPT-4o: odds ratio [OR] 239.89, 95% CI 111.05-518.01; *P*<.001; ClinicalBERT: OR 1924.07, 95% CI 687.46-5383.99; *P*<.001) and order count per study (GPT-4o: OR 1.79, 95% CI 1.38-2.31; *P*<.001; ClinicalBERT: OR 2.50, 95% CI 1.64-3.80; *P*<.001) independently predicted false positive errors in multivariate logistic regression. In subgroup analysis, all models showed reduced precision and *F*_1_ in combined-study subgroups.

**Conclusions:**

The combined radiographic report format poses distinct challenges for both radiology report quality assurance and natural language processing. The combined rule-based and GPT-4o method effectively screens for laterality errors in imbalanced real-world reports. A significant performance gap exists between balanced synthetic datasets and imbalanced real-world data. Future studies should also include real-world imbalanced data.

## Introduction

### Background

Radiographic reports play a crucial role in modern medical diagnostics. Although imaging technologies like computed tomography (CT) and magnetic resonance imaging (MRI) have advanced significantly, radiographic studies still account for the most significant proportion of examinations performed in the radiology department, with chest x-ray remaining the most common radiology study worldwide [1]. Meanwhile, errors in radiographic reports are also an important source of diagnostic errors in radiology [2], highlighting the importance of reporting quality.

Errors in radiology reports include more than missed lesions. Previous studies have systematically classified potential errors in radiology reports. For example, Kim and Mansfield [2] identified 12 primary error types, while Pinto et al [3] grouped them into 4 distinct categories. A common type of error is when radiologists identify lesions but make mistakes in interpreting them.

Laterality error is a type of interpretation error that can occur in radiology reports. This error occurs when radiologists confuse the sides of a lesion, such as mistaking the left side for the right side or vice versa. Such errors can be misleading and lead to wrong-side invasive procedures or even wrong-side surgeries [4-10]. Laterality errors have the potential to cause patient disability or even death, such as removing a patient’s only functioning kidney [11], performing a wrong-side amputation [12], or removal of the healthy, cancer-free testicle [13]. In surgical practice, wrong-side surgery is considered a “never event” [14]. For the health care system, such errors are often classified as malpractice, leading to lawsuits, compensation, and diminished trust between patients and providers [15].

Detecting laterality errors in radiographic reports can be complicated by the adaptation of combined reporting. A combined report integrates the results of multiple radiology examinations into a single assessment. For example, in breast cancer screening, combining mammography and ultrasound offers a complementary approach to cancer detection, and combining all findings into 1 report helps reduce confusion [16]. Medical institutions can also integrate radiographic studies according to their policies and requirements. For example, a trauma study could include simultaneous knee, leg, femur, and pelvis radiographs, all interpreted by 1 radiologist in 1 report, providing a comprehensive view of the patient’s injuries.

Although the combined results can be more concise for human readers to understand and communicate, they pose challenges for natural language processing (NLP), including the detection of laterality errors. Images of different anatomical parts, views, and even sides may mix in a single radiology report. Such complexity can render naive algorithms ineffective, such as checking that only “left” appears in the report or vice versa. The problem is further exacerbated by the free-typing nature of radiology reports. Radiologists have the flexibility to write combined reports without using a paragraph heading, standardized keywords, or mandatory paragraph segmentation. Currently, no literature addresses laterality errors in combined radiographic reports or their detection using NLP.

### Review of Literature

Current research on laterality errors in radiology reports is sparse. Existing research has used various algorithms to identify laterality error in radiology reports, including keyword matching or regular expression [17-19] and deep learning approaches based on ClinicalBERT [20], GPT-4 [21], and multimodal large language model (LLM) [22].

Previous studies have relied on keyword- or regular expression–based screening of text, often paired with checks against structured metadata. Sangwaiya et al [4] reported addendum-corrected laterality error rate and error rate in a separate 1-month cohort identified by simple “left or right” keyword filtering, followed by manual review. Minn et al [19] developed a keyword- and regular expression–based system that compares report text against Health Level 7 metadata to flag gender and laterality mismatches for immediate correction. Sheehan et al [18] developed an automated Structured Query Language e-trigger applied to electronic health record–derived imaging-order data, which matched structured laterality modifiers against the free-text clinical history to detect potential wrong-side orders. Landau et al [23] developed a syntax-highlighting approach that allows radiologists to review laterality-indicating terms before finalizing the report. Collectively, these approaches use relatively simple rule-based matching in structured data and free text.

Recent studies have applied deep learning and LLMs to detect errors in reports, including laterality errors. Min et al [20] used a fine-tuned ClinicalBERT [24] binary classifier to detect inconsistencies, including lesion locations, between findings and conclusions in chest x-ray reports, evaluating largely on synthetically generated errors plus a smaller, less balanced, manually edited set. Gertz et al [21] introduced 5 common error types, including side confusion, into radiographic and cross-sectional reports and found that GPT-4 underperformed the best-performing radiologist overall but was comparable to other radiologists. Kathait et al [22] used a multimodal GPT-4 family model to validate CT, MRI, and ultrasound reports flagged by commercial NLP tools for potential laterality errors, serving as secondary validation rather than a primary detector. These studies show promise of deep learning and LLMs in analyzing complex radiology reports, such as CT and MRI, but often rely on synthetic or balanced datasets, with limited evaluation as primary screening tools in real-world, imbalanced settings.

To the best of our knowledge, existing studies are limited to single-modality radiology reports and do not specifically address scenarios involving combined reports [17-23], in which different anatomical parts and laterality can mix together.

### Aim of Our Study

Our study aims to advance the detection of laterality errors in radiology reports in 3 ways. First, we define and characterize the combined radiographic report, a significant yet unstudied format in our region. We showed that combined radiographic reports are associated with a significantly higher laterality error rate and lower detection performance of state-of-the-art NLP models compared to standard noncombined reports. Second, we introduce a clinically deployable ensemble approach integrating rule-based methods with GPT-4o, rigorously evaluated on 10,000 authentic, highly imbalanced reports. Third, we quantify and contrast the performance gap between synthetic and real-world datasets and elucidate the limitations of the evaluation methodologies commonly used in prior studies.

## Methods

### Study Design

We conducted a retrospective, multisite, internal validation study using deidentified electronic health records, including radiographic reports and metadata, from 3 hospital branches between 2012 and 2024.

#### Participant Flow

Eligible studies included English radiographic reports with orders specifying laterality, and Chinese labor health examinations were excluded. The data were split into 3 datasets without overlapping: TrainVal (combined training and validation dataset), Test-1 (both real-world, imbalanced), and Test-2 (balanced, synthetic). Test-1 came from a distinct branch.

#### Intervention

All evaluations followed a fixed batch evaluation pipeline: inputs were the full radiology report and the corresponding order, processing used either rule-based methods (baseline, manual workarounds, or GPT‑4o–assisted information extraction) or pure deep‑learning comparators (fine‑tuned RoBERTa, ClinicalBERT, or GPT‑4o few‑shot), and the output was a report‑level binary prediction (positive if the report contained at least 1 laterality error).

#### Data Collection and Analysis

The primary outcomes were report-level precision, recall, and *F*_1_-score, both overall and by combined versus noncombined subgroups. Secondary analyses included qualitative error reviews and a multivariable logistic regression to identify predictors of false positives for GPT-4o and ClinicalBERT on the Test-1 and Test-2 datasets.

#### Feasibility and Acceptability Assessment

We assessed feasibility by performing end-to-end execution of our methods using both synthetic, balanced data and cross-site, real-world, imbalanced data. As a method‑oriented formative study, we did not conduct formal acceptability assessments.

### Ethical Considerations

The institutional review board at Chi Mei Hospital approved this study (11305-008). This study involves a retrospective analysis of deidentified electronic medical records (no direct identifiers were retained), eliminating the need to obtain informed consent from participants. No participant compensation was provided.

### Data Collection

We obtained the data in this study from the 3 branches of the Chi Mei hospital: the Yongkang branch (medical center, 1296 beds), the Liuying branch (regional teaching hospital, 895 beds), and the Jiali branch (regional teaching hospital, 341 beds). The 3 branches employ 25, 11, and 3 full-time radiologists, respectively. In 2024, the approximate number of radiographic studies for the 3 hospital branches was approximately 250,000, 160,000, and 70,000, respectively.

**Figure 1:**
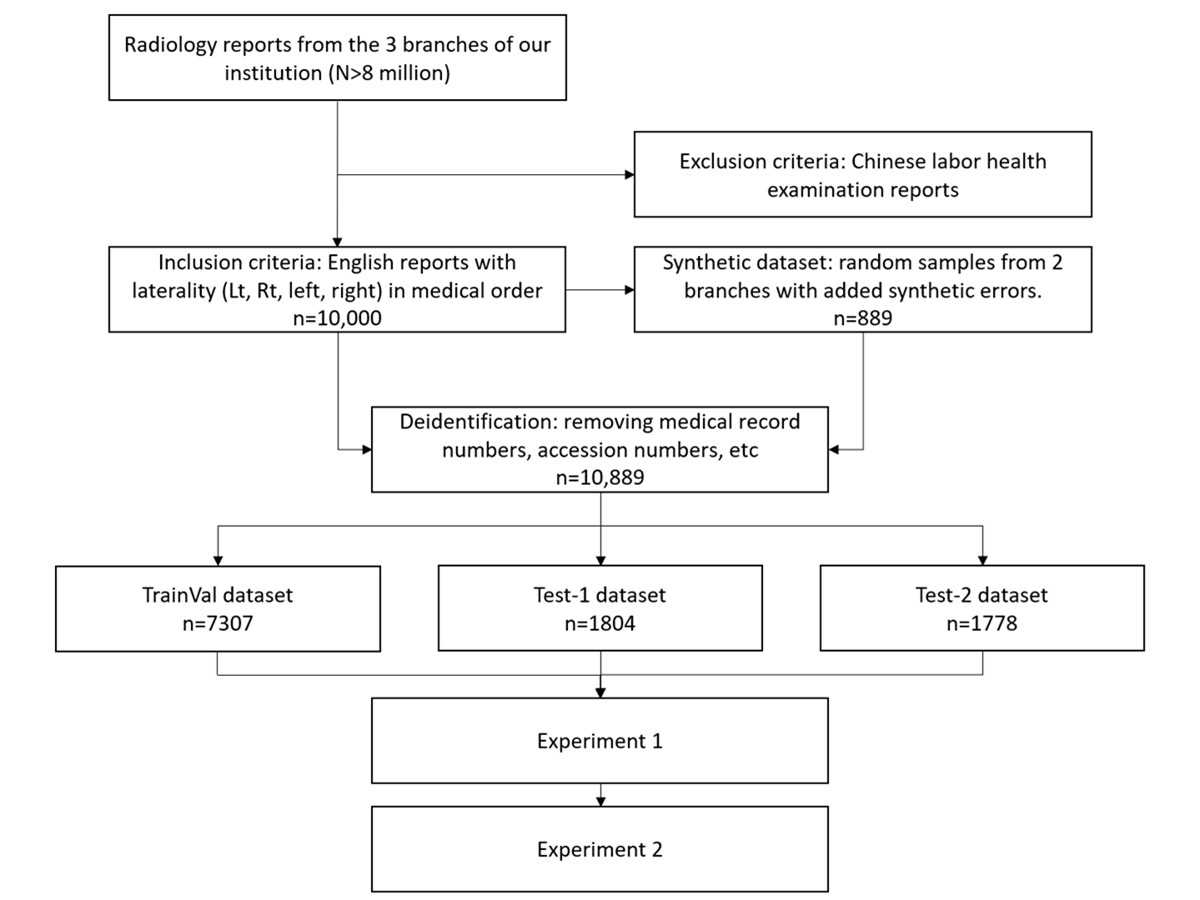
Research flow. TrainVal: combined training and validation dataset.

Figure 1 depicts the flowchart of the study. The inclusion criteria for this study include radiological examinations performed in the 3 branches of our institution from 2012 to 2024. We included radiographic (x-ray) reports that are written in English. In addition, the relevant medical orders must indicate the laterality of the imaging site using the keywords “Lt,” “Rt,” “left,” or “right.” Only cases that meet all these conditions were included. Conversely, the exclusion criteria are Chinese labor health examination reports; all cases meeting this criterion were excluded from the study.

Prior to initiating our experimental procedures, all data were subjected to a deidentification process. This involved the removal of sensitive information that could potentially identify patients, such as medical record numbers and accession numbers.

Our research includes radiographic studies with single or multiple medical orders per study. For each radiographic study that involved multiple medical orders, the radiologists identified whether the report described the findings for each order separately or combined findings related to multiple medical orders into a single paragraph.

### Dataset Preparation

#### Overview

We categorized the data into 3 datasets: TrainVal, the first test dataset (Test-1), and the second test dataset (Test-2). We used 2 test datasets to investigate the impact of different factors on the model’s performance. There is no overlap between the data in the 3 datasets.

#### TrainVal Dataset

The TrainVal datasets include data from the Yongkang and Jiali branches of the Chimei Hospital, but not from the Liuying branch.

#### Test-1 Dataset

The Test-1 dataset contains data solely from the Liuying branch to evaluate the model’s generalization capability across different hospitals and variations in physician reporting styles.

#### Test-2 Dataset

The Test-2 dataset was created with additional simulated data to improve class balancing and explore the effect of class balancing on model performance. We selected a subset of data free of laterality errors from the Yongkang and Jiali branches. KHW manually introduced errors by randomly reversing one occurrence of “Lt,” “left,” “Rt,” and “right” in the report text. These manually patched data were combined with the unmodified versions to form the Test-2 dataset. The panel of experts discussed the size of the Test-2 dataset.

### Data Annotation

A board-certified radiologist annotated all reports in this study. We did not perform double annotation or adjudication due to our sample size (10,000 real-world studies) and resource constraints. To assess interrater agreement, a second radiologist independently annotated subsamples, which we selected using label-based stratified random sampling with supersampling of the minority class, without access to the original labels or class distributions.

We focused on laterality errors that were clearly suspicious from reviewing physicians’ orders and radiology reports in accordance with our institution’s x-ray imaging protocols, such as reporting a right tibia fracture on a left knee x-ray. We classified ambiguous cases as having no laterality error because confirming them would require an image review that is beyond the scope of this study. Multimedia Appendix 1 provides examples of clearly suspicious errors.

Our research investigated discrepancies between the laterality of the lesion detailed in the reports and the laterality specified in the physicians’ orders, as well as discrepancies of laterality within the reports themselves. We do not identify errors other than laterality errors.

Our study examines the combined radiographic reports. Each report in our study could contain multiple medical orders. Some medical orders may contain multiple anatomical parts. For example, “L-S Spine anteroposterior+Lat” contains both the lumbar and sacral spines.

The format of radiographic reports is entirely free typing in our hospital. There is no requirement to use headings to indicate which medical order or anatomical part corresponds to the findings in each paragraph.

Table 1 presents a simulated example illustrating the detection of laterality errors in combined reports in this study. This example presumes the physician’s orders encompassed a chest x-ray in posteroanterior view and a left shoulder in anteroposterior view. The right clavicle fracture at line 2 is reliable and does not represent a laterality error, as a chest x-ray inherently captures both clavicles. Consequently, it is justifiable for the radiologist to document a fracture of the right clavicle. The right clavicle fracture at line 4 is incorrect, as it is in the paragraph referring to the left shoulder x-ray, which cannot depict a right clavicle fracture. The heading “Rt shoulder anteroposterior view” at line 5 represents a laterality error, as the original order indicated a left shoulder anteroposterior view, and the validity of line 6 cannot be determined.

**Table 1:** Simulated example of a combined radiographic report^a^.

Line number	X-ray report
1	Chest posteroanterior view and left shoulder anteroposterior view shows:
2	Right clavicle fracture.
3	Left shoulder anteroposterior view shows:
4	Right clavicle fracture.
5	Right shoulder anteroposterior view shows:
6	Right clavicle fracture.^b^
7	Right rib fracture.

^a^Assuming the order for this single study includes a chest x-ray posteroanterior view and a left shoulder anteroposterior view. Right clavicle at line 4 is incompatible with the left shoulder at paragraph heading at line 3, and right shoulder at line 5 is incompatible with the original order of left shoulder and are both invalid. Line 7 does not use a paragraph heading, but the right rib fracture can be explained by the chest x-ray and therefore is valid.

^b^New paragraph begins below (end of right shoulder paragraph).

### Model Development

#### Rule-Based Dictionary Look-Up Method

We implemented the baseline parser using an approach similar to a finite state machine (see Multimedia Appendix 2 for details on the parser implementation and an example). The parser scans the orders and reports term by term, identifying terms that indicate laterality, anatomy, or findings. It then checks for laterality based on the scanned terms and the expert rules.

We encoded our expert rules as key-value pairs in a dictionary format. The dictionary key represents an anatomical part, while the value is a list of possible body parts or findings associated with that part. For example, the key “knee” includes the values “femur” and “tibia,” while the key “chest” includes “clavicle” and “ribs.” Values in different keys may overlap; for example, “Fibula” may appear in values of keys “knee” and “leg.”

Radiologist KHW manually curated and refined the expert rules based on TrainVal results (see Multimedia Appendix 2 for examples of expert rule modification). For each false positive, the radiologist identified and adjusted problematic terms in the expert rules, such as removing the term or assigning it to the appropriate anatomical part. For each false negative, the radiologist reviewed the report text to identify missed errors and updated the rules with relevant terms.

To reduce the expert workload, automated methods were used to generate initial drafts of expert rules, which the experts then manually refined and validated. We segmented reports into paragraphs using keyword-matched subheadings (eg, lines ending with a colon) and inferred the relevant anatomical part for each section from subheading keywords (eg, “chest” and “knee”). After lemmatizing and removing stop words with Natural Language Toolkit, we identified potential laterality-dependent terms by extracting words from 2 words before and 3 words after laterality indicators (eg, “Lt,” “Rt,” and “bilateral”). These terms, paired with their respective anatomical parts, formed key-value pairs for the draft expert rules.

Besides the baseline version, we implemented 2 additional approaches to improve the rule-based method: first, using GPT-4o information extraction for report preprocessing, and second, having experts create workarounds in the parser based on individual physician writing styles.

In the first approach, GPT-4o was used to extract anatomical locations, such as the names of bones, joints, and organs, along with the laterality from radiology reports, standardize terminology, and remove extraneous, potentially confounding details. We also instructed the GPT-4o to express all results in singular nouns whenever possible.

We hypothesize that using GPT for anatomical information extraction can standardize expressions across various styles of report texts, thereby enhancing the robustness of expert rules. For instance, terms such as “Rt genu valgum,” “Rt genu varum,” and even misspelled versions like “genuvarum, Rt.” and “genuvalgum, Rt.” can be accurately interpreted by GPT as pertaining to the right knee and captured by the same expert rule of “knee.” After extracting anatomical information from the reports using GPT-4o, we used this extracted information as parser input when appropriate.

In the second approach, KHW analyzed the TrainVal report dataset to identify common nonstandard expressions, specifically inverted writing styles such as “genuvarum, Rt,” and create specific workarounds in the parser. These workarounds may not apply to other hospitals or radiologists.

#### Fine-Tuned RoBERTa and ClinicalBERT With Noisy, Automatically Generated Labels

In this experiment, we approached the task as a sentence contradiction task. We used radiologists’ reports and combined them with medical orders, with the view information removed, leaving only the anatomical part, to form sentence pairs. We mapped the laterality errors as contradictions in the sentences. If there is no laterality error, it is considered a noncontradiction and vice versa. In addition to the gold labels annotated by radiologists, we included automatically generated samples with laterality errors in the model training to ensure proper class balancing.

For each report in the training data, we patched 1 of every 2 words indicating left and right, such as “Lt” and “Rt,” alternatively, changing from left to right and vice versa. This modification can create inconsistencies between the paragraph text and the heading and between the paragraph heading and the medical orders. The training program labeled these automatically generated samples as having laterality errors and included them in the training data.

We used the RoBERTa large model fine-tuned on the Multi-Genre Natural Language Inference corpus, which focuses on the sentence contradiction task and evaluates a model’s ability to determine whether a premise sentence entails, contradicts, or is neutral concerning a hypothesis sentence [25]. The hyperparameters used in this study are as follows: the learning rate is 2×10^–5^, the batch size for training per device is 16, and the batch size for evaluation per device is also 16. The Adam optimizer was used with the weight decay parameter set to 0.01. We capped training at 60 epochs and used early stopping: training stopped if the validation metric failed to improve for 3 consecutive epochs. We used 20% of the TrainVal for validation.

For ClinicalBERT, the hyperparameters are as follows: the learning rate is 5×10^–5^, and the batch size for training and evaluation per device is 32. All other hyperparameters remain consistent with those used in the RoBERTa experiments.

#### GPT-4o With Few-Shot Prompting

In our experiments with LLMs, we used GPT-4o and approached the task as a binary text classification problem with few-shot and chain-of-thought prompting [26] and in-context learning. The task of the LLM was to categorize each report as either containing or not containing laterality errors.

We provided examples for assessing laterality errors, including abstract conceptual and 4 concrete examples [27] using simulated reports and physician orders. We provided the radiologist’s hand-crafted step-by-step reasoning alongside responses for each example. We required the model to use the chain-of-thought technique and output its step-by-step reasoning process, reflecting the examples provided by the radiologist in the prompt, regardless of the final prediction of the model.

#### Common Model Implementation Details

The models were optimized solely based on the TrainVal, without using the Test-1 or Test-2 datasets, including the creation and optimization of algorithms and dictionaries for expert rule-based methods, the training data for the RoBERTa and ClinicalBERT models, and the radiology reports referenced for GPT prompting.

To improve the GPT’s performance and reproducibility of the results, we used the following methodology in all cases: first, we instructed the GPT to role-play as a radiologist [28]. Next, we specified the abbreviations and conventions commonly used in our institution. Finally, we set the temperature to 0 during the application programming interface call and used a fixed seed of 5566.

The view information was removed, leaving only the anatomical part whenever the models needed to refer to medical order information in the experiments, either through chain-of-thought reasoning guided by our prompts in the GPT-4o with few-shot prompting experiment or via dictionary 1-way mapping in other experiments. For example, different views of a chest x-ray, such as posteroanterior, lordotic, oblique, and decubitus, were all simplified to “chest.”

This design greatly simplified the creation of expert rules for the expert rule-based methods. Rules only need to be created for each anatomical part without considering different combinations of views. This approach forced the deep learning methods to focus on the anatomical part rather than the view information.

### Evaluation

#### Overview

We frame laterality error detection as a binary text classification task. For each x-ray study, the model ingests the full radiology report together with all associated medical orders and predicts whether at least 1 laterality error is present; a single study may be linked to one or more orders. The rule-based method additionally requires expert rules. The model outputs whether the report contains any laterality error.

We conducted 2 experiments.

#### First Experiment

In the first experiment, we developed a rule-based dictionary look-up method and compared it with 2 optimized versions using hand-crafted workarounds and GPT-4o information extraction for text preprocessing.

#### Second Experiment

In the second experiment, we compared the method with the best recall from the first experiment with pure deep learning approaches, including fine-tuned RoBERTa and ClinicalBERT with noisy labels and GPT-4o with few-shot prompting.

We conducted subgroup analyses to compare model performance in noncombined and combined studies. Test-1 and Test-2 data were merged to avoid complete class absence in specific subgroups while maintaining the imbalanced distribution characteristic of real-world data. We reported primary metrics separately for each test set and used the combined data only for subgroup analyses.

We gathered all cases, in which ClinicalBERT made highly confident predictions (softmax probability≥0.9) that were incorrect. Using the transformer-interpret package, the 5 most attributed words per case were identified. A board-certified radiologist then reviewed these words for relevance to laterality errors.

We additionally used multivariate logistic regression models to investigate the factors underlying the performance differences in false positive prediction between ClinicalBERT and GPT-4o across the Test-1 and Test-2 datasets after adjusting for potential confounders.

### Statistical Analysis

We used Python (version 3.12.3; Python Software Foundation) with SciPy (version 1.12.0) and scikit-posthocs (version 0.11.3) for statistical analysis. The significance level (α) was set at .05, and all analyses were 2-tailed unless otherwise specified.

#### Demographics

We compared error rates and anatomical part distributions among TrainVal, Test-1, and Test-2 using chi-square tests. All observations were independent. For error rates, all expected cell counts exceeded 5. For analysis of anatomical parts, we merged temporomandibular joint and neck categories, resulting in all expected counts exceeding 1, with approximately 2.47% (2/81) of cells below 5. We used Bonferroni correction for pairwise comparisons (α=.0167) and reported Cramér *V* as the effect size.

A 2-proportion *z* test was used to compare laterality error rates between combined and noncombined reports. We verified that the expected number of events and nonevents per group exceeded 10 (based on the pooled proportion). Errors in reports were independent.

#### First Experiment

In the first experiment, we used the Friedman test to compare the recall ranks of the baseline and 2 optimized rule-based dictionary look-up methods. For each of the 3 datasets, the recall for each method was computed, after which the methods were ranked within each dataset (rank 1=highest recall).

After finding significant differences, we used the Nemenyi post-hoc test to identify which versions differed. The nonparametric, repeated measures rank data and paired observations of the 3 methods across the 3 datasets satisfy the assumptions of both tests. Scikit-posthocs provides direct access to pairwise *P* values, eliminating the need to calculate critical differences for the Nemenyi test manually.

#### Second Experiment

In the second experiment, multivariate logistic regression models were additionally used to investigate the factors underlying the false positive prediction of ClinicalBERT and GPT-4o across the Test-1 and Test-2 datasets. We constructed separate logistic regression models for the evaluation of ClinicalBERT and GPT-4o, with each observation corresponding to a positive prediction made by the model (ClinicalBERT or GPT-4o) on a sample from either dataset (Test-1 or Test-2). All observations were independent, with no duplicates within each analysis.

The 2 primary independent variables were dataset membership (Test-1 or Test-2) and order count per examination. The models included anatomical location (ankle, femur, finger, foot, forearm, hip, humerus, leg, scapula, shoulder, wrist, and others) as covariates. Anatomical part variables are not mutually exclusive; each anatomical part covariate is included in the model as a separate binary variable indicating presence or absence in the observation. The outcome variable indicates whether a prediction was a false positive. All independent variables are binary, except for the order count per examination, which is a continuous variable. We performed the sensitivity analysis using the *E* value [29] to evaluate the robustness of our findings to potential unmeasured confounding, with the calculation based on the observed odds ratio (OR) point estimate for rare outcome (<15%) or the square root of OR otherwise. Due to the imbalance in the outcome variable, we applied frequency weights to prevent the dominant class from skewing the results.

The following diagnostic checks were performed to ensure that our model met the assumptions of logistic regression. The outcome variable was defined as a dichotomous variable. We excluded binary independent variables with fewer than 10 observations in the least frequent category. We checked for linearity of the continuous covariate (order count per examination) using the Box-Tidwell test. We retained outliers identified using Cook distance (4/N) and standardized residuals (|*z*|>3) because they reflected valid data. Boolean OR was used to combine specific dichotomous covariates for anatomical locations into an “others” category, reducing multicollinearity, decreasing the variance inflation factor, and improving model stability. As our focus is on the association between the primary independent variables and the outcome rather than the specific impact of each anatomical location, the combination is justified.

## Results

### Demographics

Table 2 shows the demographic information of the datasets. As of July 2024, our study includes 10,889 studies comprising 10,000 real-world radiographic studies from 3 hospital branches corresponding to 20,635 orders, 22,523 distinct anatomical parts, and 889 synthetic studies containing manually generated errors. The anatomical parts were derived by removing the view information and laterality from the original medical orders. For example, “Rt knee anteroposterior+lateral+Merchant view, L-spine anteroposterior+lateral view” was simplified to “Knee, L-spine.”

**Table 2:** Demographics.

			TrainVal^a^		Test-1	Test-2		Test-2 (before augmentation)		*P* value^b^	
**Studies, n**			7307		1804	1778		889		<.001	
	Without laterality error	7205		1786		889	889				
	With laterality error	102		18		889	0				
Attending physicians, n			27		12	27		27		—^c^	
Orders, n			14,399		4503	3466		1733		—	
Orders per study, mean (SD)			1.97 (0.97)		2.50 (0.86)	1.95 (1.02)		1.95 (1.02)		<.001	
**Anatomical parts involved, n (%)^d^**											<.001
	Abdomen	174 (1.11)		56 (1.13)		2 (0.05)	1 (0.05)				
	Ankle	1023 (6.54)		298 (6)		274 (7.16)	137 (7.16)				
	Cervical spine	241 (1.54)		103 (2.07)		64 (1.67)	32 (1.67)				
	Calcaneus	62 (0.40)		16 (0.32)		18 (0.47)	9 (0.47)				
	Chest	2228 (14.24)		762 (15.35)		344 (8.99)	172 (8.99)				
	Clavicle	357 (2.28)		111 (2.24)		104 (2.72)	52 (2.72)				
	Coccyx	44 (0.28)		7 (0.14)		6 (0.16)	3 (0.16)				
	Elbow	601 (3.84)		221 (4.45)		158 (4.13)	79 (4.13)				
	Femur	528 (3.38)		188 (3.79)		128 (3.34)	64 (3.34)				
	Finger	108 (0.69)		8 (0.16)		32 (0.84)	16 (0.84)				
	Foot	1029 (6.58)		293 (5.90)		246 (6.43)	123 (6.43)				
	Forearm	226 (1.44)		57 (1.15)		86 (2.25)	43 (2.25)				
	Hand	893 (5.71)		283 (5.70)		260 (6.79)	130 (6.79)				
	Hip	368 (2.35)		188 (3.79)		106 (2.77)	53 (2.77)				
	Humerus	234 (1.50)		74 (1.49)		56 (1.46)	28 (1.46)				
	Knee	2035 (13.01)		577 (11.62)		506 (13.22)	253 (13.22)				
	Lumbar spine	611 (3.91)		194 (3.91)		184 (4.81)	92 (4.81)				
	Leg	579 (3.70)		156 (3.14)		158 (4.13)	79 (4.13)				
	Lower limb	37 (0.24)		1 (0.02)		4 (0.10)	2 (0.10)				
	Neck	6 (0.04)		7 (0.14)		4 (0.10)	2 (0.10)				
	Pelvis	999 (6.39)		427 (8.6)		248 (6.48)	124 (6.48)				
	Sacrum	542 (3.46)		149 (3)		162 (4.23)	81 (4.23)				
	Scapula	126 (0.81)		18 (0.36)		30 (0.78)	15 (0.78)				
	Shoulder	1144 (7.31)		375 (7.55)		304 (7.94)	152 (7.94)				
	Skull	141 (0.90)		44 (0.89)		24 (0.63)	12 (0.63)				
	Thoracic spine	125 (0.80)		55 (1.11)		30 (0.78)	15 (0.78)				
	Temporomandibular joint	3 (0.02)		1 (0.02)		0 (0)	0 (0)				
	Wrist	1180 (7.54)		296 (5.96)		290 (7.58)	145 (7.58)				

^a^TrainVal: combined training and validation dataset.

^b^Comparison for TrainVal, Test-1, and postaugmentation Test-2 datasets.

^c^Not available.

^d^Anatomical parts are obtained by removing view information from order (eg, chest x-ray posteroanterior becomes chest); each combined report may correspond to multiple anatomic parts.

Of the 10,000 real-world, nonsynthetic studies, 3000 contain only 1 medical order (noncombined studies). Among 7000 studies containing multiple orders (combined studies), 4325 reported each order’s findings separately, and 2675 merged findings in reporting, with the results corresponding to multiple medical orders combined into a single paragraph.

The error rates of the 3 datasets differed significantly (*χ*^2^_2_=4193.9; *P*<.001; Cramér *V*=0.62). Bonferroni-adjusted post hoc tests (α=0.0167) revealed no significant difference between TrainVal and Test-1 (*χ*^2^_1_=1.5; *P*=.23). All comparisons with artificially balanced Test-2 remained highly significant (*P*<.001), reflecting its intentional difference in class ratio.

The anatomical site distributions across the 3 datasets (TrainVal, Test-1, and Test-2) were statistically significantly different (*χ*^2^_52_=322.7; *P*<.001). However, the effect size was small (Cramér *V*=0.081), reflecting only minimal practical differences in distributions.

### Distribution of Laterality Errors

The real-world data collected contains 120 cases of laterality errors, resulting in an error rate of 1.20% (120/10,000). Of these cases, 97.50% (117/120) contain 1 laterality error per report, and 2.50% (3/120) contain 2 errors per report.

The laterality error rate was 1.47% (103/7000) for combined reports compared to 0.57% (17/3000) for noncombined reports. The difference in proportions was 0.90% (95% CI 0.52%-1.29%), which was statistically significant (*z*=3.81; *P*<.001, 2-proportion *z* test).

### Experiment 1

#### Primary Performance Metrics

Table 3 shows the impact of different optimization strategies on the rule-based dictionary look-up method. The rank of recall of 3 versions of the rule-based method shows significant differences (*Q*=6.0; *P*=.0498; *df*=2, Friedman test). The version enhanced with GPT information extraction (average rank=1) achieved the highest recall across all datasets, significantly outperforming the baseline (average rank=3; *P*=.04, Nemenyi test). There was no significant difference in recall between the GPT and workaround versions (average rank=2; *P*=.43, Nemenyi test).

**Table 3:** Impact of optimization strategies on the performance of rule-based methods.

		Precision	Recall	*F*_1_-score
**TrainVal^a^ dataset**				
	Baseline	0.885	0.676	0.767
	+Workaround^b^	*0.912^c^*	0.912	*0.912*
	+GPT-4o^d^	0.874	*0.951*	0.911
**Testing (Test-1 dataset)**				
	Baseline	0.833	0.556	0.667
	+Workaround	*0.875*	0.778	*0.824*
	+GPT-4o	0.696	*0.889*	0.780
**Testing (Test-2 dataset)**				
	Baseline	0.992	0.882	0.934
	+Workaround	0.993	0.897	0.942
	+GPT-4o	*0.996*	*0.925*	*0.959*

^a^TrainVal: combined training and validation dataset.

^b^Workarounds: implement additional workarounds for individual radiologists’ grammatically incorrect free-text reports.

^c^The top result for each metric is reported in italics format.

^d^GPT-4o: use GPT-4o to extract information before applying the rule-based method.

#### Subjective Error Analysis of Combined Rule-Based Method With GPT-4o

The rule-based method combined with GPT information extraction yielded 9 incorrect predictions (7 false positives and 2 false negatives) on Test-1. Contralateral report comparison contributed to 55.55% (5/9) of errors, misconfigured expert rules accounted for 22.22% (2/9), and other unaddressed scenarios in algorithm design—such as dictating the same side twice in a bilateral report—made up 22.22% (2/9) of the errors. None of the errors resulted from the omission of view information or terms misattributed to unrelated orders.

GPT information extraction reduced 2 false negatives but added 6 false positives in the Test-1 dataset compared to the workaround version. One of the reduced false negatives resulted from GPT’s terminology standardization, where plural terms were converted to singular forms so that expert rules could correctly capture them. In total, 5 of the 6 additional false positives were due to limitations in the parser design rather than errors in the GPT information extraction. In these cases, radiologists simultaneously mentioned both the left and right sides for comparison, which was intentional and not a laterality error. In the experiment of a rule-based parser with a workaround, the expert rules coincidentally lacked plural versions of keywords, leading to error cancellation that masked these hidden errors. The parser could trigger these false positives only after the GPT information extraction standardized the terms.

### Experiment 2

#### Primary Performance Metrics

Table 4 compares the rule-based method enhanced with GPT information extraction and those based solely on deep learning. The rule-based method enhanced with GPT information extraction consistently performed well on both imbalanced, real-world TrainVal and Test-1 datasets and the balanced, synthetic Test-2 dataset. In the balanced, synthetic Test-2 dataset, both rule-based methods enhanced with GPT information extraction and GPT-4o performed well.

ClinicalBERT, trained on data from the artificial error generator, achieved a recall of 0.667 on the Test-1 dataset, detecting only two-thirds of laterality errors. RoBERTa achieved a recall of 0.000, suggesting that it did not effectively learn from the error patterns.

ClinicalBERT trained with noisy labels and GPT-4o using few-shot prompting showed substantial performance degradation when applied to the 2 real-world, imbalanced datasets, especially in terms of precision.

**Table 4:** Performance of different methods with subgroup analysis.

		Precision	Recall	*F*_1_-score
**TrainVal^a^ dataset**				
	Rule-based+GPT-4o^b^	*0.874^c^*	*0.951*	*0.911*
	GPT-4o^d^	0.423	0.892	0.574
	RoBERTa^e^	0.500	0.010	0.019
	ClinicalBERT^f^	0.142	0.843	0.244
**Testing (Test-1 dataset)**				
	Rule-based+GPT-4o	*0.696*	*0.889*	*0.780*
	GPT-4o	0.219	*0.889*	0.352
	RoBERTa	0.000	0.000	0.000
	ClinicalBERT	0.047	0.667	0.088
**Testing (Test-2 dataset)**				
	Rule-based+GPT-4o	0.996	0.925	0.959
	GPT-4o	0.979	*0.953*	*0.966*
	RoBERTa	*1.000*	0.007	0.013
	ClinicalBERT	0.984	0.749	0.851
**Test-1+Test-2 (noncombined subgroup)**				
	Rule-based+GPT-4o	*1.000*	0.929	0.963
	GPT-4o	0.997	*0.943*	*0.969*
	RoBERTa	*1.000*	0.017	0.034
	ClinicalBERT	0.966	0.798	0.874
**Test-1+Test-2 (combined subgroup)**				
	Rule-based+GPT-4o	*0.981*	0.921	*0.950*
	GPT-4o	0.878	*0.957*	0.916
	RoBERTa	0.000	0.000	0.000
	ClinicalBERT	0.619	0.715	0.664

^a^TrainVal: combined training and validation dataset.

^b^Rule-based+GPT-4o: combined rule-based dictionary look-up and GPT-4o extraction.

^c^The top result for each metric is reported in italics format.

^d^GPT-4o: GPT-4o with few-shot prompting.

^e^RoBERTa: fine-tuned RoBERTa with noisy, automatically generated labels.

^f^ClinicalBERT: fine-tuned ClinicalBERT with noisy, automatically generated labels.

#### Subgroup Analysis

Subgroup analysis showed that all models had lower precision and *F*_1_-scores in the combined-study subgroups than in the noncombined-study subgroups.

#### Multivariable Logistic Regression for Predictors of False Positives in ClinicalBERT and GPT-4o

Table 5 summarizes the multivariable logistic regression results. We found that order count per examination or anatomical part differences cannot explain away the effect of data membership (in Test-1) on increased false positive predictions for GPT-4o and ClinicalBERT.

**Table 5:** Multivariate logistic regression analysis examining the associations between false positive prediction errors of the GPT-4o and ClinicalBERT models on the Test-1 and Test-2 datasets and the characteristics of combined reports.

Variable		GPT-4o		ClinicalBERT	
		OR^a^ (95% CI)	*P* value	OR (95% CI)	*P* value
In Test-1		239.89 (111.05-518.01)	<.001	1924.07 (687.46-5382.99)	<.001
Order count		1.79 (1.38-2.31)	<.001	2.50 (1.64-3.80)	<.001
**Anatomical parts**					
	Ankle	1.89 (0.97-3.67)	.06	0.90 (0.24-3.35)	.88
	Femur	3.31 (1.69-6.49)	<.001	0.53 (0.09-3.05)	.48
	Finger	8.72 (2.54-29.90)	<.001	0.95 (0.01-71.59)	.98
	Foot	1.25 (0.63-2.48)	.52	0.43 (0.11-1.75)	.24
	Forearm	0.27 (0.06-1.26)	.10	1.21 (0.24-6.25)	.82
	Hip	3.21 (1.52-6.75)	<.001	0.17 (0.02-1.32)	.09
	Humerus	0.19 (0.03-1.03)	.05	0.90 (0.15-5.53)	.91
	Leg	2.01 (1.04-3.88)	.04	0.43 (0.10-1.86)	.26
	Scapula	2.77 (0.74-10.46)	.13	0.51 (0.00-86.14)	.80
	Shoulder	0.90 (0.45-1.82)	.77	0.23 (0.06-0.89)	.03
	Wrist	1.24 (0.59-2.61)	.57	0.88 (0.27-2.92)	.84
	Others	0.86 (0.42-1.77)	.68	2.84 (0.66-12.24)	.16

^a^OR: odds ratio.

After adjusting for anatomical parts, both Test-1 membership and order count remained significant independent predictors of false positive errors for GPT-4o (Test-1: OR 239.89, 95% CI 111.05-518.01; *P*<.001; order count: OR 1.79, 95% CI 1.38-2.31; *P*<.001) and ClinicalBERT (Test-1: OR 1924.07, 95% CI 687.46-5382.99; *P*<.001; order count: OR 2.50, 95% CI 1.64-3.80; *P*<.001). Associations for anatomical parts were variable and inconsistent across models. We included these variables primarily for covariate adjustment rather than causal inference.

*E* value sensitivity analysis indicated substantial robustness to unmeasured confounding (GPT-4o: in Test-1: 479.29, order count: 2.97; and ClinicalBERT: in Test-1: 87.2, order count: 2.53).

#### Subjective Error Analysis of ClinicalBERT on the Test-1 Dataset

**Figure 2:**
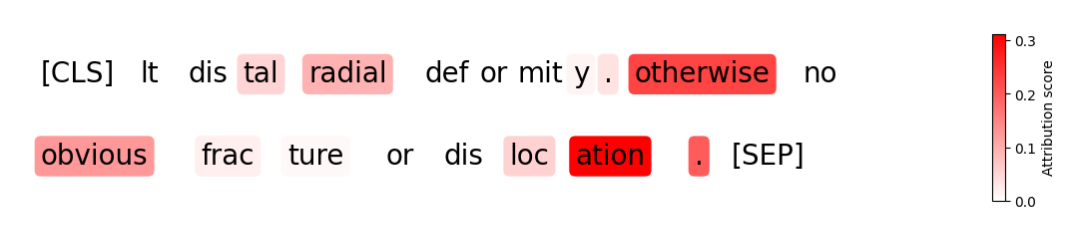
A high-confidence false-positive prediction from ClinicalBERT (softmax probability≥0.9). Corresponding orders were anteroposterior and lateral views of the bilateral ankles, wrists, and cervical spine. Word attribution analysis shows that ClinicalBERT overlooked important keywords, such as “Lt” and assigned attention to irrelevant words, such as “otherwise” and “obvious.”.

Figure 2 shows a representative example of word attribution analysis. Of the 44 high-confidence (softmax probability≥0.9) incorrect predictions (38 false positives and 6 false negatives), we identified the 5 most attributed words per case, totaling 220 words. Only 46.82% (103/220) of these words were relevant to laterality error assessment. Therefore, even in high-confidence cases, ClinicalBERT often relies on features unrelated to the target task.

#### Subjective Error Analysis of GPT-4o on the Test-1 Dataset

In our analysis of GPT-4o’s wrong predictions on the Test-1 dataset, 47.46% (28/59) resulted from misclassifying valid reporting styles, such as splitting or merging descriptions of both sides or legitimate omission, as laterality errors. Additionally, 18.64% (11/59) of incorrect predictions involved misclassifying nonlaterality errors (eg, partial order omissions) as laterality errors, 15.25% (9/59) reflected inconsistent or self-contradictory reasoning (eg, stating that no laterality error was present while still flagging the case as positive), and 11.86% (7/59) reflected a mismatch between the anatomical part of the findings and the clinical orders. Report quality issues accounted for 3.39% (2/59), while discrepant but legitimate error classifications and false negatives each comprised 1.69% (1/59). Multimedia Appendix 3 presents an example for each type of error, accompanied by GPT-4o’s reasoning and our explanation.

### Interrater Agreement in Data Annotation

We selected 500 samples for independent annotation, including 100 of 120 samples with laterality errors identified by the main annotator and 400 of 9880 samples without laterality errors. Independent annotation by a second annotator yielded a κ value of 0.998, with disagreement in one case, which the main annotator had labeled as positive.

## Discussion

### Principal Findings

#### Distinct Challenges Presented by Combined Reports

Combined reports pose distinct challenges for radiology report quality assurance. Our results show a significantly higher laterality error rate in combined reports compared to noncombined reports.

In addition to increased reporting errors, combined reports also present new challenges for NLP. In experiment 2, models that perform well on noncombined reports, such as GPT-4o and ClinicalBERT, exhibit substantial drops in performance on the combined reports subgroup, particularly in precision (Table 4). Furthermore, multivariable logistic regression (Table 5) identifies the order count per study as an independent predictor of false positive errors for both models, even after adjusting for covariates. Our findings show that although methods for noncombined reports remain applicable, their effectiveness declines in the context of combined reports.

#### Performance Analysis of Pure Deep Learning–Based Methods

Our results point to class imbalance as a potentially important factor in reducing the precision of pure deep learning methods. Both GPT-4o and ClinicalBERT demonstrated much lower precision on the imbalanced Test-1 dataset compared to the balanced Test-2 dataset, while recall remained similar—a pattern consistent with the behavior seen in imbalanced datasets dominated by negative samples (samples without laterality errors). Furthermore, in our multivariate logistic regression analysis (Table 5), the order count per study and anatomical distribution did not fully account for the increased false positives. Sensitivity analysis indicated substantial robustness of our results to unmeasured confounding. These findings support class imbalance as the primary explanation for the observed reduction in precision.

ClinicalBERT trained with artificial error generator data achieved high precision (>0.9) on the balanced, synthetic Test-2 dataset while attaining moderate recall on both the Test-1 (0.667) and Test-2 (0.749) datasets. These results suggest that the synthetic errors from our error generator used for training partially overlap with real-world error patterns, enabling the model to identify a subset of clinically relevant cases. However, these artificial errors fail to capture the full diversity and complexity of real-world laterality errors. Our word attribution analysis (Figure 2) further revealed that only 46.82% (103/220) of the top 5 most attributed words were relevant to laterality error detection, indicating that ClinicalBERT may rely on spurious correlations or shortcut features unrelated to the target task.

These results underscore both the value and the limitations of synthetic data augmentation. While our error generator can supplement model training with clinically plausible erroneous patterns, it cannot fully substitute for diverse, real-world cases. Expanding and diversifying the training data, especially by incorporating realistic clinical errors or improving the diversity of synthetically generated samples, will be essential for achieving robust and generalizable model performance.

Additionally, the consistently superior performance of ClinicalBERT over RoBERTa in terms of recall across all datasets demonstrates that domain-specific pretrained models better capture relevant patterns in our screening task.

#### Role and Limitations of the Synthetic Test-2 Dataset

We use the synthetic, class-balanced Test-2 as a controlled diagnostic dataset and rely on the real-world, imbalanced, cross-site Test-1 for deployment-facing conclusions. By design, Test-2 was created manually by flipping exactly 1 laterality term per report, matching the per-report error count in 97.50% (117/120) of real-world positive cases. Such random flips can produce contradictions across orders, subheadings, and paragraph text. We did not attempt to align the distribution of error contexts with heterogeneous real-world reporting conditions (eg, missing subheadings, merged descriptions in combined studies, legitimate contralateral comparisons, and difficult-to-align anatomies), nor did we model reports with multiple laterality errors or nonrandom-flip error patterns (eg, copy-paste with an unedited side), including patterns we may not have identified. Thus, while Test-2 captures single-term lexical substitutions, it does not fully capture the more complex structural contexts and error etiologies observed in practice, which can alter task difficulty and interfere with model performance. Consistent with these differences, all models showed lower recall on Test-1 than on Test-2 in experiment 2 (Table 4), suggesting that Test-1 may contain more challenging cases that are underrepresented in Test-2.

Therefore, our results suggest that while synthetic data grounded in real-world observations can yield optimistic estimates of model performance on common, easily observed laterality error manifestations, it cannot entirely replace real-world evaluation, given its constrained scope and the distributional differences from real reports. Future work could broaden the scope of synthetic patterns and expand real-world, nonsynthetic cases to more accurately assess model performance in clinical settings.

#### Proposed Solution for Laterality Error Screening in Combined Radiographic Reports

We found that the combination of GPT-4o with rule-based methods shows strong performance in real-world screening. The integration of GPT-4o significantly improves the recall of the rule-based parser (Table 3), while maintaining acceptable sensitivity and overall performance. In Test-1, the GPT-optimized version had 6 more false positives compared to the workaround version, mostly (5/6) due to parser limitations triggered by GPT. Thus, GPT extraction did not significantly affect precision, and our hybrid approach improves case detection with minimal loss of specificity in real-world screening.

**Figure 3:**
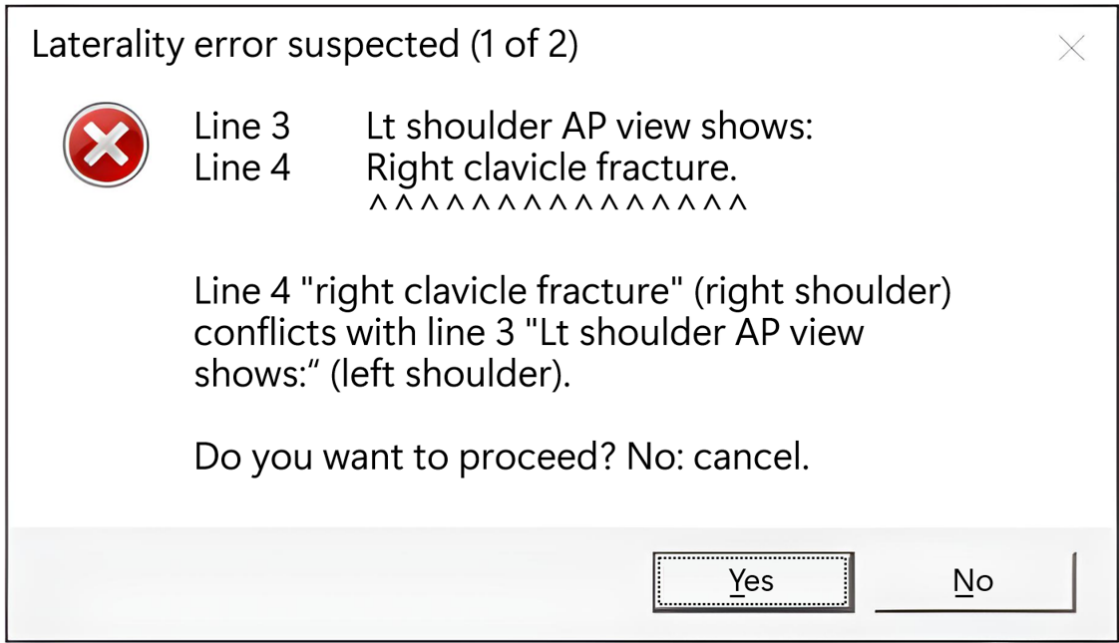
A conceptual message box for detecting laterality errors using our method for the example in Table 1. Line 4 is incompatible with the paragraph heading at line 3, which is left shoulder only. Our method permits a human-readable error message with line-level alignment among the problematic sentences, the GPT-extracted entities (not shown), and the parser-mapped anatomical parts with laterality.

Our method can be embedded into the radiology reporting workflow as a presignature, soft‑block quality assurance step within the radiology information system. Figure 3 illustrates a conceptual message box after integration. When a radiologist attempts to finalize a report, the client can call a backend web service with the order information and the full draft report. Our methods allow line-level alignment among the original sentences, the GPT-extracted entities, and the parser-mapped anatomical parts and laterality. The user interface can present returned results with inline highlighting and 1-click options to revise or proceed with finalization. All alerts, GPT and parser outputs, user actions, and the versions of the ruleset, prompt, and model can be stored to support reproducibility, root‑cause analysis of false alerts, and retrospective review.

In daily use, this review-before-release check aims to reduce preventable harm by flagging likely laterality errors prior to sign-off, without adding undue burden to the workflow, given the performance of our method on real-world, imbalanced data. For safety improvement beyond prospective use, batch analysis of historical reports can detect previously missed laterality errors to enable timely correction and remediation. For sites that cannot deploy LLMs due to cost or privacy constraints, a rule‑based parser with hand‑crafted workarounds is a practical fallback; however, its effectiveness relies on continuous implementation modifications (not only rule updates) to keep pace with shifting local reporting styles over time, imposing an ongoing maintenance burden. By contrast, our combined rule-based parser and GPT-4o approach standardizes heterogeneous language upstream automatically, leading to improved recall and reduced manual maintenance efforts simultaneously, resulting in a more sustainable and scalable clinical NLP pipeline.

### Comparison to Prior Work

Table 6 provides a comparative summary of related studies that have used NLP methods to detect laterality errors.

**Table 6:** Comparison of studies using natural language processing (NLP) methods to detect laterality errors.

	NLP algorithm	Laterality comparison	Values, n	Synthetic data	Partial label validation
Lee et al [17]	Keyword matching	Examination name, report	300	Yes	No
Lee et al [17]	Keyword matching	Examination name, report	29,257	No	No
Sheehan et al [18]	Keyword matching	Image side, clinical history or order	76,468	No	Yes
Minn et al [19]	Keyword matching regular expression	HL7^a^ metadata, impression of report	391,657	No	Yes
Min et al [20]	Fine-tuned ClinicalBERT	Finding, impression of report	187,673	Yes	No
Kathait et al [22]	Multimodal GPT-4	Finding, impression of report, image	898	No	No
Gertz et al [21]	GPT-4	Finding and impression of report	200	Yes	No
Our study	Ruled-based method with or without GPT, RoBERTa, GPT-4o	Order, full report	10,000^b^	No	No
Our study	Ruled-based method with or without GPT, RoBERTa, GPT-4o	Order, full report	886^b^	Yes	No

^a^HL7: Health Level 7.

^b^Combined report included.

Our study is the first to define and systematically evaluate the combined report as a distinct category of complex radiology reports. By evaluating both existing and newly proposed NLP methods for these reports, we identified and addressed unique challenges that have not been previously reported. Furthermore, we demonstrated a significant performance gap between commonly used synthetic, balanced datasets and large-scale, real-world imbalanced data, underscoring the importance of real-world evaluation for the clinically robust deployment.

Although previous studies by Gertz et al [21] and Kathait et al [22] have also focused on complex reports, such as CT and MRI, they did not address the challenges posed by the combined report format or real-world evaluation using highly imbalanced data. In addition, Kathait et al [22] evaluated the performance of the multimodal GPT-4 in a second look at data flagged as laterality errors by commercial NLP tools, in contrast to our study’s focus on first-line screening.

Sheehan et al [18] and Minn et al [19] used a more significant number of real-world samples, but did not fully evaluate all included data for laterality errors. They only assessed whether the data identified as positive by the NLP algorithm were true or false positives. Similar to other research [18-22], the study by Lee et al [17] does not address the complexities that arise from combined reports. Their study, published in 2015, also lacks an evaluation of modern approaches based on deep learning and LLMs.

### Future Directions

#### Algorithmic Improvement for Rule-Based Method

Our results show that expert rule-based approaches are not without limitations, as experts may not consider all possible exceptions when designing algorithms and formulating expert rules. For example, we did not consider the scenario in the Test-1 dataset where radiologists intentionally referenced the opposite side for comparison, resulting in false positive predictions.

Despite the effectiveness of our method on real-world data, these edge cases reveal opportunities for further improvement in clinical deployment. Addressing potential mismatches between the development and test data and improving data diversity can help experts identify issues early in the development process. Algorithmic refinements such as context-aware postprocessing may also further enhance the robustness of our approach. Our findings underscore the importance of ongoing optimization and iterative expert review to ensure real-world applicability and support the continued adaptation to the evolving clinical environment.

Automatic extraction of expert rules is also a viable direction for future research. The significant manual effort to analyze samples and formulate rules limits the application of rule-based methods. If automated methods could support the extraction of expert rules, rule-based approaches would become significantly more feasible.

Finally, our method still assumes specific reporting styles (eg, using “shows:” as a subheading); thus, external adaptation requires prior analysis of local reporting conventions. Future work could focus on enhancing the parser’s flexibility to accommodate heterogeneous reporting formats better and improve generalizability across institutions.

#### Future LLM Applications

Currently, LLMs have shown varying degrees of success in many radiology-related applications. The systematic review by Keshavarz et al [30] categorized ChatGPT applications in radiology into 5 broad clinical domains. Notably, ChatGPT excels at simplifying complex radiology reports into more straightforward, more structured formats. However, LLMs also have well-documented limitations and potential pitfalls, such as difficulties with the complex, multistep reasoning tasks that are often required in advanced applications [31].

Our study encountered notable difficulties in GPT-4o reasoning. In experiment 2, most (55/59, 93.22%) GPT-4o’s errors on the Test-1 dataset were reasoning-related, including misclassification of valid reporting styles or nonlaterality errors as laterality errors, inconsistent or self-contradictory reasoning, and mismatched anatomical parts. These results highlight limitations in GPT-4o’s reasoning capabilities in our task. To address these difficulties, we divided the task into anatomical parts extraction using LLMs and matching with traditional algorithms, achieving significantly better performance than using LLMs alone. Based on previous literature [30] and our experimental results, we infer that our hybrid approach is more effective because it leverages GPT’s strengths in transforming and summarizing reports into clearer formats while mitigating its weaknesses in the complex multistep reasoning required for our task.

This strategy may provide insights for applications beyond the detection of laterality errors. Rather than relying on LLMs for all tasks end-to-end, future NLP applications may achieve better performance by using LLMs for language understanding and assigning high-precision, context-dependent, algorithmic subtasks, such as logical decision-making for rare error screening, to more accurate methods. Such modular approaches use the complementary strengths of each method and may enable broader applications in NLP.

#### Future Studies Using Deep Learning for Laterality Error Detection

Our study shows that high performance on class-balanced, synthetic datasets does not guarantee similar outcomes in real-world, imbalanced scenarios where clinical deployment ultimately occurs. Deep learning methods such as GPT-4o and ClinicalBERT performed well on our balanced Test-2 dataset, a finding similar to the results of Min et al [20] and Kathait et al [22]. However, their performance severely degraded in the real-world imbalanced Test-1 dataset. The findings of Min et al [20] also showed a downward trend in the area under the precision-recall curve, precision, and recall of the model’s performance in imbalanced datasets compared to more balanced datasets. We also found that real-world data may contain more challenging, underrepresented cases, whereas controlled synthetic data may capture only more obvious error patterns. These findings raise concerns about relying solely on balanced or synthetic datasets for benchmarking and about the real-world performance of pure deep learning methods.

Therefore, we recommend that future studies additionally include imbalanced real-world datasets without simulated samples into their evaluation frameworks, moving beyond synthetic, balanced samples. Our results demonstrate that real-world validation, which accurately reflects the clinical environment, is essential for developing robust and reliable clinical NLP applications suitable for deployment in actual workflows.

### Limitations of Our Study

Our study has several limitations. First, our method for detecting laterality errors references the laterality of medical orders without the original images. Thus, our method cannot identify some laterality errors, such as mislabeling a left rib fracture as a right rib fracture on a chest x-ray. Our metadata-based method also cannot entirely eliminate false positives, as we cannot fully exclude the possibility that some studies may include minimal contralateral-side images. However, both laterality error detection based on metadata and images of studies are important and represent different but complementary approaches.

Second, several factors may affect the comparability of our study with other studies. For example, our research compares physician orders and free-text reports rather than findings and impressions. In addition, our study does not include more complex CT, MRI, or ultrasound reports. The varying complexity of free-text reports could influence the performance of the methods. However, our research is clinically relevant because radiographic reports are among the most common studies in practice, and discrepancies between the laterality of free text and physician orders are a major source of laterality errors. Our institution does not have combined CT or MRI studies available for research.

Third, the quantity and proportion of data with laterality errors are limited in real-world datasets, meaning that even minor differences in model predictions can significantly impact performance metrics. Nevertheless, our team has collected sufficient samples to the best of our ability, and our study included class-balanced samples for performance evaluation, similar to previous research.

In addition, we have not addressed all the known issues of the expert rule-based models. Our initial attempts to add rules for edge cases increased system complexity and reduced reliability due to unintended rule interactions. Introducing test-specific fixes could also affect unbiased evaluation of the model’s performance on new data. Despite such limitations, the current results show that the rule-based model applies to this problem.

Moreover, given the large sample size (10,000 real-world studies), resource limitations, and the pilot or feasibility nature of our study, only 1 board-certified radiologist performed all primary annotations. Single-annotator designs risk subtle systematic bias such as same-directional handling of borderline or ambiguous cases, intrarater drift, and fatigue-related undercalling and incorporation bias, as the same radiologist defined the guidelines and expert rules. Our study mitigated incorporation bias by grounding a high-level guideline—including an a priori definition of ambiguity—in imaging standards rather than providing detailed instructions specific to anatomy-view combinations and finalizing it before evaluation. We developed expert rules only on TrainVal and froze them before evaluating model performances on Test‑1 and Test‑2. For label reliability, an independent radiologist—uninvolved in guideline or rule design—double‑annotated a stratified subsample enriched for the minority class based on their own knowledge of imaging standards, blinded to primary labels, model outputs, and class prevalence. Agreement was near‑perfect (Cohen κ=0.998). Nonetheless, with only 2 raters, the possibility of residual common-direction bias cannot be completely excluded, especially for ambiguous cases.

Furthermore, our rule-based method does not use view information. While the rule-based approach combined with GPT-4o exhibited no errors attributable to missing view information in Test-1, we acknowledge that, due to the complexity of x-ray imaging, view information may be essential for laterality error detection in some instances.

Finally, our study is an internal validation study, and we selected Test-1 from a different institution. We cannot exclude the possibility that unquantified data drift or systematic institutional differences not included in our analysis (eg, reporting styles or clinical practices) may also exert their effects through data membership, together with the effect of class imbalance. Our sensitivity analysis cannot exclude these factors. Our approach does not substitute for stratified splitting at the partitioning stage to control data drift and ensure unbiased model development and evaluation. Further studies with larger and more diverse external datasets address these issues. Nonetheless, cross-site validation with different branches directly demonstrates model performance in a new clinical setting, which is crucial for real-world applications.

### Conclusions

Combined radiographic reports pose distinct challenges for both clinical radiology report quality assurance and NLP. The ensemble method that integrates GPT-4o information extraction with expert rule-based methods detects laterality errors effectively in combined radiographic reports on real-world, highly imbalanced data. Carefully and manually optimized rule-based methods without GPT-based information extraction are also practical alternatives.

Our study found a significant performance gap between the results using well-balanced augmented data and real-world highly imbalanced data. We suggest additional inclusion of imbalanced real-world datasets without simulated samples in future studies.

## Appendix

Multimedia Appendix 1 Examples of clearly suspicious laterality errors in x-ray of the knee.

Multimedia Appendix 2 The parser implementation details of rule-based dictionary look-up method.

Multimedia Appendix 3 One representative example of each error type produced by GPT-4o on the Test-1 dataset, together with GPT-4o’s reasoning and our analysis.

## Abbreviations

CT: computed tomography
LLM: large language model
MRI: magnetic resonance imaging
NLP: natural language processing
OR: odds ratio
TrainVal: combined training and validation dataset

## Data Availability

The software code, expert rules for the rule-based method, fine-tuned ClinicalBERT and RoBERTa models, and GPT prompts in this study are available in the author KHW’s repository [27,32]. The deidentified patient data are not publicly available due to institutional policies but are available from the corresponding author on reasonable request.
