# Supplementary material for: Detecting Laterality Errors in Combined Radiographic Studies by Enhancing the Traditional Approach With GPT-4o: Algorithm Development and Multisite Internal Validation
Source: JMIR Form Res. 2025 Oct 29;9:e76384. doi: 10.2196/76384 (PMC12612642; doi:10.2196/76384)
Supplement: Multimedia Appendix 1 [file formative_v9i1e76384_app1.docx]

When obtaining an anteroposterior (AP) and lateral view X-ray of the knee, one should center the imaging field over the knee joint. The radiograph must encompass the distal portion of the femur, the knee joint itself, as well as the proximal regions of both the tibia and fibula [33].

Given these criteria, the following descriptions are considered to have no evidence of laterality errors when applied to radiographic images labeled as left knee AP and lateral views:

1. Left distal femur fracture.
2. Comminuted fracture of the left patella.
3. Osteoarthrosis of the left knee.

Conversely, the following descriptions highly suggest a laterality error under the same imaging circumstances:

1. Right distal femur fracture.
2. Comminuted fracture of the right patella.
3. Osteoarthrosis of the right knee.
