# Supplementary material for: Detecting Laterality Errors in Combined Radiographic Studies by Enhancing the Traditional Approach With GPT-4o: Algorithm Development and Multisite Internal Validation
Source: JMIR Form Res. 2025 Oct 29;9:e76384. doi: 10.2196/76384 (PMC12612642; doi:10.2196/76384)
Supplement: Multimedia Appendix 2 [file formative_v9i1e76384_app2.docx]

## Baseline Parser Design

We implemented the baseline rule-based parser using a method similar with a finite state machine. The finite state machine contains two critical states: current laterality and current scope.

The current laterality has four possible states: Any, Left, Right, and Both. The parser reset the state to Any at the beginning of each new line. The parser proceeds word by word, switching states when it encounters laterality-indicating words such as "left," "right," "bilateral," or "both." If the parser encounters a non-laterality word, the laterality of the word is determined based on the value of the current laterality.

The current scope consists of a global and a local mode. When the parser fails to detect a substring that indicates a subheading (such as "shows:") within a paragraph, the parser works in global mode. In global mode, for each word requiring a laterality check, the parser performs a reverse dictionary look-up against expert rules to find keys that have the word as a value and are related to the medical orders of the study. This process generates a list of candidate anatomical parts potentially related to the word and their correct laterality. The parser checks if the correct laterality of any candidate explains the current laterality of the word; if not, it raises a laterality error. If a heading restricts the paragraph to a subset of medical orders or anatomical parts, the parser first checks for laterality errors in the heading, then switches to local mode and focuses only on the mentioned subset.

## Walkthrough of Example

To improve intuition, we explain the abbreviated process by which the parser would identify laterality errors in the example shown in Table 1 in the Methods section.

At line 1, the term "shows:" indicates a paragraph heading. The term "left" changes current laterality and modifies the following words including “shoulder”, and left shoulder is consistent with the order. The parser then switches to local mode and use the key chest and left shoulder in the expert rules.

At line 2, the term "right" changes the current laterality and modifies words including "clavicle." The parser notes that both the values of key chest and the shoulder include “clavicle”, with the "right clavicle" explained by the chest. The parser switches back to global mode at the end of the paragraph.

At line 3, the “shows:” indicates heading again, and the heading suggests that the paragraph only describes the left shoulder, not the chest X-ray. The parser switches to local mode and only use the shoulder key of expert rules. The “clavicle” in line 4 appears within values of the shoulder key. However, the laterality “right” does not match the correct laterality of the shoulder “left” in the study, indicating a laterality error. The parser does not look at the chest key in this paragraph.

At line 5, the "shows:" indicates a paragraph heading . The "right" modifies words including "shoulder." The mention of the right shoulder does not match the medical order and indicates a laterality error in the heading.

At line 7, the parser does not detect paragraph heading, operates in global mode, and uses all keys relevant to the orders, including chest and shoulder, of the expert rules. The parser determines that the right ribs can be explained by the chest.

## Examples Illustrating Parser Rule Modification

We present two simulated examples to demonstrate how errors in expert rules can lead to false positives and false negatives. In Table 1 in the Methods section, both the Chest and Shoulder keys in the expert rules must explicitly include the term “clavicle” to avoid such errors.

If the Chest key omits “clavicle,” the parser generates a false positive in Line 2 by incorrectly attributing “clavicle” solely to the Shoulder context. This triggers an error because “right clavicle” and “left shoulder” refer to opposite sides. Including “clavicle” under the Chest key allows the parser to recognize that it can also pertain to the chest, thereby preventing this error.

Conversely, if the Shoulder key excludes “clavicle,” a false negative occurs in Line 4, as the parser does not associate any term with laterality and thus fails to trigger the intended error. Adding “clavicle” to the Shoulder key ensures the parser recognizes its dependency on laterality, enabling accurate error detection.
