# Supplementary material for: Detecting Laterality Errors in Combined Radiographic Studies by Enhancing the Traditional Approach With GPT-4o: Algorithm Development and Multisite Internal Validation
Source: JMIR Form Res. 2025 Oct 29;9:e76384. doi: 10.2196/76384 (PMC12612642; doi:10.2196/76384)
Supplement: Multimedia Appendix 3 [file formative_v9i1e76384_app3.docx]

| Misclassifying valid reporting styles as laterality errors. | |
| --- | --- |
| Order | Wrist Lt PA+Lat  Wrist Rt PA+Lat |
| Report | - No definite bony fracture.  - No joint dislocation or subluxation. |
| Result | False Positive |
| GPT-4o's reasoning | The order specifies: right and left wrist. The report does not specify laterality for the wrist. Since the order includes both right and left wrist, the report should specify laterality to ensure both sides are covered. The absence of laterality in the report creates ambiguity and potential laterality error. |
| Our assessment | Legitimate omission of subheading, not an error. |
|  | |
| Misclassifying other errors as laterality errors | |
| Order | Leg Lt AP+Lat  Knee Lt AP+Lat  Foot Rt AP+oblique |
| Report | Lt knee and leg:  - No definite bony fracture.  - No joint dislocation or subluxation. |
| Result | False Positive |
| GPT-4o's reasoning | The order specifies: right foot, left leg, and left knee. The report describes: left knee and leg, but does not mention the right foot. The laterality of the foot is missing in the report, which is a laterality error because the right foot was part of the order. |
| Our assessment | Part of the orders (Foot Rt AP+oblique) was erroneously neglected, not a laterality error. |
|  | |
| Faulty or self-contradictory reasoning | |
| Order | Knee Lt AP+Lat  Foot Rt AP+oblique  Skull Zygomatic Arch |
| Report | Left knee, right foot, skull zygomatic arch:  No bony fracture or bony pathology.  No joint subluxation or dislocation. |
| Result | False Positive |
| GPT-4o's reasoning | The order specifies: right foot, left knee, and skull zygomatic arch. The report describes: left knee, right foot, and skull zygomatic arch. The laterality of the knee in the report (left) matches the order (left), and the laterality of the foot in the report (right) matches the order (right). There is no laterality error in the report. |
| Our assessment | GPT labeled the case as positive, despite stating that "There is no laterality error in the report." |
|  | |
| Mismatching between the anatomical part of findings and clinical orders | |
| Order | Leg Lt (Include Knee)  Clavicle bilateral AP  C-Spine AP+Lat  Pelvis AP |
| Report | Pelvis, C-spine, bilateral clavicle, left leg:  - Left scapula and ribs fracture.  - Suspected left fibular shaft linear fracture.  - Degeneration change with spur formation of C-T-L spine.  - Generalized diminished bone density. |
| Result | False Positive |
| GPT-4o's reasoning | The order specifies: C-Spine, bilateral clavicle, pelvis, and left leg (including knee). The report mentions a left scapula fracture, which is not included in the order. The scapula is not part of the ordered study, and its laterality (left) does not match any ordered study. There is a laterality error because the report includes findings on a part not ordered. |
| Our assessment | GPT failed to recognize that a bilateral clavicle X-ray also includes the scapula. |
|  | |
| Report quality issues | |
| Order | Elbow Lt AP+Lat  Wrist Lt PA+Lat |
| Report | Lt wrist:  - No definite bony fracture.  - No joint dislocation or subluxation.  Lr wlbow:  Anterior fat pad sign, probalby due to lipohemoarthrosis. occult fracture can not be rule out. |
| Result | False Positive |
| GPT-4o's reasoning | The order specifies: left elbow and left wrist. The report (Lr wlbow:) describes: right elbow. The laterality of elbow in report (right) is different from the order (left). There is a laterality error. |
| Our assessment | GPT misidentified the typo "Lr wlbow" as "right elbow," resulting in further errors. |
|  | |
| Discrepant but legitimate error classifications | |
| Order | Leg Lt AP+Lat  Knee Rt AP+Lat |
| Report | AP + lateral views of both knees:  No bony fracture or bony pathology.  No joint subluxation or dislocation. |
| Result | False Positive |
| GPT-4o's reasoning | The order specifies: right knee and left leg. The report describes: both knees. The laterality of knee in report (both) is different from the order (right). There is laterality error. |
| Our assessment | The report’s errors allow two reasonable interpretations: GPT identified a laterality error by miscoding "Rt knee" as "both knees," while we considered it a miscoding of "leg" as "knee." |
|  | |
| False negatives | |
| Order | Wrist Rt PA+Lat  Hand Rt PA+Oblique |
| Report | Hand Rt PA+Oblique:  Symmetric subluxation of bilateral 1st carpometacarpal joints on PA views, in favor of normal variant.  No bony pathology or recent fracture.  Wrist Rt PA+Lat:  No bony pathology or recent fracture. |
| Result | False Negative |
| GPT-4o's reasoning | The order specifies: right wrist and right hand. The report (Hand Rt PA+Oblique: Wrist Rt PA+Lat:) describes: right hand and right wrist. The laterality of hand and wrist (right) is the same as the order (right). There is no laterality error. |
| Our assessment | All PA views included in this exam—hand and wrist—were for the right side, rather than one for each side, preventing comparison of the bilateral first carpometacarpal joints. |
